# 3,3′-(*p*-Phenyl­enedimethyl­ene)di­imidazol-1-ium bis­(3-carb­oxy-4-hydroxy­benzene­sulfonate) dihydrate

**DOI:** 10.1107/S1600536809002086

**Published:** 2009-01-23

**Authors:** Yong-Li Peng, Li-Hui Jia

**Affiliations:** a School of Materials Science and Engineering, Wuhan Institute of Technology, Wuhan 430073, People’s Republic of China

## Abstract

In the title compound, C_14_H_16_N_4_^2+^·2C_7_H_5_O_6_S^−^·2H_2_O, the 3,3′-(*p*-phenyl­enedimethyl­ene)diimidazol-1-ium dication lies on a crystallographic inversion center. In the crystal structure, dications, anions and solvent water mol­ecules are linked *via* O—H⋯O, N—H⋯O and C—H⋯O hydrogen bonds, and C—H⋯π inter­actions, forming a three-dimensional network containing *R*_2_^2^(4), *R*_2_^4^(12), *R*_4_^4^(22), *R*_8_^10^(32) and *R*_12_^14^(66) ring motifs.

## Related literature

For information on hydrogen-bond graph-set motifs, see: Bernstein *et al.* (1995 ▶). For the synthesis and crystal structure of 1,4-bis­(imidazol-1-ylmeth­yl)benzene, see: Hoskins *et al.* (1997 ▶). For related crystal structures, see: Meng *et al.* (2007 ▶, 2008 ▶); Muthiah *et al.* (2003 ▶); Smith *et al.* (2004 ▶, 2005*a* ▶,*b* ▶,*c* ▶).
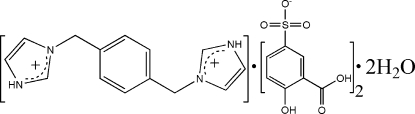


## Experimental

### 

#### Crystal data

C_14_H_16_N_4_^2+^·2C_7_H_5_O_6_S^−^·2H_2_O
*M* *_r_* = 710.68
Triclinic, 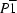

*a* = 7.9975 (6) Å
*b* = 8.8060 (7) Å
*c* = 11.2419 (8) Å
α = 90.784 (1)°
β = 96.656 (1)°
γ = 97.342 (1)°
*V* = 779.61 (10) Å^3^
*Z* = 1
Mo *K*α radiation
μ = 0.25 mm^−1^
*T* = 292 (2) K
0.20 × 0.10 × 0.10 mm

#### Data collection

Bruker SMART APEX CCD area-detector diffractometer
Absorption correction: multi-scan (*SADABS*; Sheldrick, 1997 ▶) *T*_min_ = 0.942, *T*_max_ = 0.976
8434 measured reflections
3168 independent reflections
2524 reflections with *I* > 2σ(*I*)
*R*_int_ = 0.024

#### Refinement

*R*[*F* ^2^ > 2σ(*F* ^2^)] = 0.050
*wR*(*F* ^2^) = 0.142
*S* = 1.12
3168 reflections
232 parameters
H atoms treated by a mixture of independent and constrained refinement
Δρ_max_ = 0.34 e Å^−3^
Δρ_min_ = −0.24 e Å^−3^

### 

Data collection: *SMART* (Bruker, 2001 ▶); cell refinement: *SAINT-Plus* (Bruker, 2001 ▶); data reduction: *SAINT-Plus*; program(s) used to solve structure: *SHELXS97* (Sheldrick, 2008 ▶); program(s) used to refine structure: *SHELXL97* (Sheldrick, 2008 ▶); molecular graphics: *PLATON* (Spek, 2003 ▶); software used to prepare material for publication: *PLATON*.

## Supplementary Material

Crystal structure: contains datablocks global, I. DOI: 10.1107/S1600536809002086/lh2757sup1.cif

Structure factors: contains datablocks I. DOI: 10.1107/S1600536809002086/lh2757Isup2.hkl

Additional supplementary materials:  crystallographic information; 3D view; checkCIF report

## Figures and Tables

**Table 1:** Hydrogen-bond geometry (Å, °) *Cg*1 is the centroid of atoms N1/N2/C12–C14.

*D*—H⋯*A*	*D*—H	H⋯*A*	*D*⋯*A*	*D*—H⋯*A*
O1—H1⋯O7^i^	0.93 (4)	1.68 (4)	2.593 (3)	168 (3)
O3—H3*A*⋯O2	0.87 (2)	2.038 (19)	2.591 (3)	121 (3)
O3—H3*A*⋯O3^ii^	0.87 (2)	2.46 (2)	2.883 (4)	111 (2)
O7—H7*B*⋯O4^iii^	0.81 (4)	1.93 (4)	2.741 (3)	173 (4)
O7—H7*A*⋯O5	0.88 (4)	1.93 (4)	2.799 (3)	172 (4)
N1—H1*A*⋯O6	0.79 (3)	1.95 (3)	2.707 (3)	160 (3)
C4—H4⋯O6^iv^	0.93	2.51	3.411 (4)	164
C12—H12⋯O2^v^	0.93	2.22	3.035 (3)	146
C14—H14⋯O6^vi^	0.93	2.52	3.219 (4)	132
C3—H3⋯*Cg*1^vii^	0.93	2.97 (1)	3.889 (3)	170

Symmetry codes: (i) 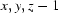; (ii) 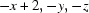; (iii) 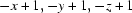; (iv) 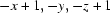; (v) 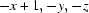; (vi) 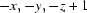; (vii) 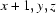.

**Fig. 1.**
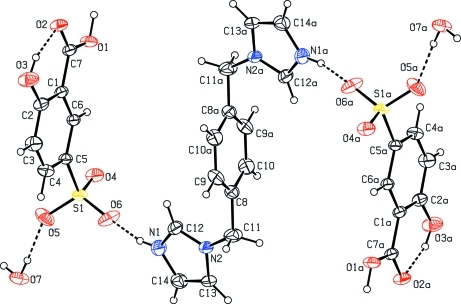
The molecular structure of (I), showing the atom-numbering scheme. Displacement ellipsoids are drawn at the 30% probability level and H atoms are shown as small spheres of arbitrary radii. Atoms marked with 'a' are at position -x, -1 - y, -z. The H-bonds are shown as dashed lines.

**Fig. 2.**
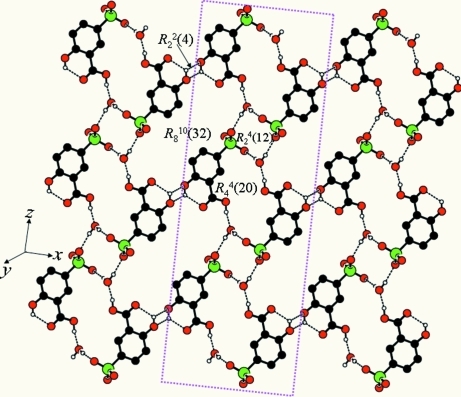
Part of the crystal structure of (I), showing the formation of the two-dimensional sheet running parallel to (110) plane. Hydrogen bonds are shown as dashed lines. Hydrogen atoms not involved in the motif have been omitted for clarity. The outlined area shows the one-dimensional tape running parallel to the [001] direction.

**Fig. 3.**
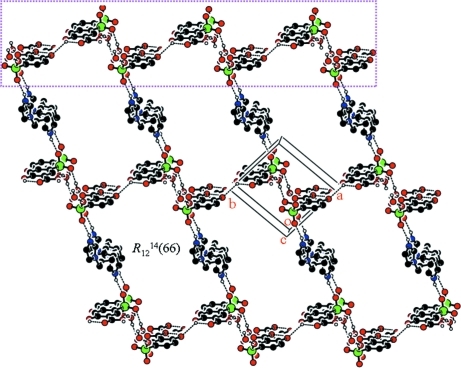
Part of the crystal structure of (I), showing the formation of the three-dimensional network. Hydrogen bonds are shown as dashed lines. Hydrogen atoms not involved in the motif have been omitted for clarity. The outlined area shows the two-dimensional wrinkled sheet parallel to the (110) plane.

## supplementary crystallographic information

### Comment

5-Sulfosaliyclic acid (5-H_2_SSA), a strong organic acid (p*K*_a1_=2.6), can easily release its sulfonic hydrogen atom to a nitrogen-containing Lewis bases (Muthiah *et al.*, 2003; Smith *et al.*, 2004, 2005*a*, 2005*b*, 2005*c*; Meng *et al.*, 2007; Meng *et al.*, 2008), forming organic salts in most cases. In this paper, we report the crystal structure of the title compound, C_14_H_16_N_4_.2(C_7_H_5_O_6_S).2(H_2_O) (I) , which was obtained by crystallization of the 95% methanol solution of 5-H_2_SSA and 1,4-Bis(imidazol-1-ylmethyl)benzene) (bix) (molar ratio 2:1) at room temperature.

In (I), the asymmetric unit consists of one half bix^2+^ dication, one complete 5-HSSA^-^ anion and one water molecule (see Fig. 1 for symmetry complete formula unit). Similar to some analogs (Meng *et al.*, 2007, 2008), the hydrogen atom was transferred from the sulfonic group to the imidazole nitrogen atom, forming an 1:2 organic salt (cation to anion). The hydroxyl O3 atom forms an intramolecular *S*(6) and an intermolecular *R*_2_^2^(4) ring, resulting in a 5-HSSA^- ^ dimer (Bernstein, *et al.*, 1995). The carboxyl O1 atom is only hydrogen-bonded to a water molecule at (*x*, *y*, *z* - 1). The plane defined by sulfonic O4/O5/O6 atoms makes a dihedral angle of 88.7 (1)° with the C1—C6 plane, with the distances of each oxygen atom away from the benzene-plane being 0.211 (1), 1.126 (1) and 1.237 (1) Å, respectively, which is slightly different from those recently reported by Meng, *et al.*, 2007, 2008. In the bix^2+ ^ dication, there is an crystallogrphic inversion centre at the centre of gravity of the benzene ring.

In the crystal structure of (I), the ionic components are linked by a cooperative action of N—H···O, O—H···O and C—H···O hydrogen bonds into a continuous three-dimensional network which is further consolidated by a C—H···π interaction (Table 1). In more detail, the supramolecular structure in (I) can be analyzed in terms of three substructures. First, by utilizing three intermolecular O1—H1···O7^i ^ [symmetry code: (i) *x*, *y*, *z* - 1], O7—H7A···O5 and O7—H7B···O4^ii^ [symmetry code: (ii) 1 - *x*, 1 - *y*, 1 - *z*] hydrogen bonds, the 5-HSSA^-^ anions and water molecules are linked into a one-dimensional tape structure running parallel to the [001] direction (Fig.2) in which alternating *R*_2_^4^(12) and *R*_4_^4^(20) (Bernstein, *et al.*, 1995) hydrogen-bonded rings are formed. Secondly, these adjacent one-dimensional tapes are further joined together by the hydroxylic *R*_2_^2^(4) hydrogen-bonded ring, resulting in a two-dimensional wrinkled sheet running parallel to the (110) plane. In addition to, the intramolecular S(6) and intermolecular *R*_2_^2^(4) hydrogen-bonded rings, another *R*_8_^10^(32) hydrogen-bond motif is formed in the sheet (Fig.2). These sheets are joined by means of the N1—H1A···O6 hydrogen bond, forming a three-dimensional network (Fig.3) in which larger *R*_12_^14^(66) hydrogen-bonded rings are observed. Further analysis (using the program PLATON [Spek, 2003]) shows that these three-dimensional networks are strengthened by C—H···O hydrogen bonds and weak C—H···π interactions  (Table 1).

### Experimental

1,4-Bis(imidazol-1-ylmethyl)benzene) (bix) was prepared according to a literature method (Hoskins *et al., *1997). 1:2 molar amounts of bix (47.6 mg, 0.2 mmol) and 5-sulfosalicylic acid dihydrate (101.6 mg, 0.4 mmol) were dissolved in 95% methanol (20 ml). The mixture was stirred for 30 min at ambient temperature and then filtered. The resulting colourless solution was kept in air for one week. Block colourless crystals of (I) suitable for single-crystal X-ray diffraction analysis were grown at the bottom of the vessel by slow evaporation of the solution (yield 86.4 mg, 60.8%, based on a 1:2 salt).

### Refinement

H atoms bonded to C atoms were positioned geometrically with C–H = 0.93Å (aromatic) and 0.97Å (methylene) and refined in a riding-model approximation  *U*_iso_(H) = 1.2*U*_eq_(C). H atoms bonded to O and N atoms were found in difference Fourier maps and the N—H and O—H distances were refined freely [the refined distances are given in Table 1; *U*_iso_(H) = 1.2*U*_eq_(N) and 1.5*U*_eq_(O)].

### Crystal data

**Table:**

C_14_H_16_N_4_^2+^·2C_7_H_5_O_6_S^−^·2H_2_O	*Z* = 1
*M**_r_* = 710.68	*F*(000) = 370
Triclinic, *P*1	*D*_x_ = 1.514 Mg m^−^^3^
Hall symbol: -P 1	Mo *K*α radiation, λ = 0.71073 Å
*a* = 7.9975 (6) Å	Cell parameters from 2637 reflections
*b* = 8.8060 (7) Å	θ = 2.3–28.0°
*c* = 11.2419 (8) Å	µ = 0.25 mm^−^^1^
α = 90.784 (1)°	*T* = 292 K
β = 96.656 (1)°	Block, colorless
γ = 97.342 (1)°	0.20 × 0.10 × 0.10 mm
*V* = 779.61 (10) Å^3^	

### Data collection

**Table:**

Bruker SMART APEX CCD area-detector diffractometer	3168 independent reflections
Radiation source: fine focus sealed Siemens Mo tube	2524 reflections with *I* > 2σ(*I*)
graphite	*R*_int_ = 0.024
0.3° wide ω exposures scans	θ_max_ = 26.5°, θ_min_ = 1.8°
Absorption correction: multi-scan (*SADABS*; Sheldrick, 1997)	*h* = −9→10
*T*_min_ = 0.942, *T*_max_ = 0.976	*k* = −11→11
8434 measured reflections	*l* = −12→14

### Refinement

**Table:**

Refinement on *F*^2^	Primary atom site location: structure-invariant direct methods
Least-squares matrix: full	Secondary atom site location: difference Fourier map
*R*[*F*^2^ > 2σ(*F*^2^)] = 0.050	Hydrogen site location: inferred from neighbouring sites
*wR*(*F*^2^) = 0.142	H atoms treated by a mixture of independent and constrained refinement
*S* = 1.12	*w* = 1/[σ^2^(*F*_o_^2^) + (0.0671*P*)^2^ + 0.3146*P*] where *P* = (*F*_o_^2^ + 2*F*_c_^2^)/3
3168 reflections	(Δ/σ)_max_ < 0.001
232 parameters	Δρ_max_ = 0.34 e Å^−^^3^
0 restraints	Δρ_min_ = −0.24 e Å^−^^3^

### Special details

**Table:**

Geometry. All e.s.d.'s (except the e.s.d. in the dihedral angle between two l.s. planes) are estimated using the full covariance matrix. The cell e.s.d.'s are taken into account individually in the estimation of e.s.d.'s in distances, angles and torsion angles; correlations between e.s.d.'s in cell parameters are only used when they are defined by crystal symmetry. An approximate (isotropic) treatment of cell e.s.d.'s is used for estimating e.s.d.'s involving l.s. planes.
Refinement. Refinement of *F*^2^ against ALL reflections. The weighted *R*-factor *wR* and goodness of fit *S* are based on *F*^2^, conventional *R*-factors *R* are based on *F*, with *F* set to zero for negative *F*^2^. The threshold expression of *F*^2^ > σ(*F*^2^) is used only for calculating *R*-factors(gt) *etc*. and is not relevant to the choice of reflections for refinement. *R*-factors based on *F*^2^ are statistically about twice as large as those based on *F*, and *R*- factors based on ALL data will be even larger.

### Fractional atomic coordinates and isotropic or equivalent isotropic displacement parameters (Å^2^)

**Table:**

	*x*	*y*	*z*	*U*_iso_*/*U*_eq_	
C1	0.6276 (3)	0.1488 (3)	0.0830 (2)	0.0345 (5)	
C2	0.7379 (3)	0.0442 (3)	0.1231 (2)	0.0411 (6)	
C3	0.7390 (4)	−0.0098 (3)	0.2385 (3)	0.0498 (7)	
H3	0.8114	−0.0803	0.2645	0.060*	
C4	0.6339 (4)	0.0403 (3)	0.3140 (2)	0.0460 (6)	
H4	0.6351	0.0032	0.3911	0.055*	
C5	0.5250 (3)	0.1465 (3)	0.2763 (2)	0.0341 (5)	
C6	0.5211 (3)	0.1995 (3)	0.1617 (2)	0.0333 (5)	
H6	0.4474	0.2693	0.1362	0.040*	
C7	0.6258 (3)	0.2045 (3)	−0.0404 (2)	0.0367 (6)	
C8	−0.0498 (4)	−0.5225 (3)	0.1136 (2)	0.0445 (6)	
C9	0.0677 (4)	−0.6056 (3)	0.0730 (2)	0.0501 (7)	
H9	0.1143	−0.6774	0.1221	0.060*	
C10	−0.1170 (4)	−0.4163 (3)	0.0397 (3)	0.0517 (7)	
H10	−0.1963	−0.3591	0.0661	0.062*	
C11	−0.1021 (5)	−0.5466 (4)	0.2371 (3)	0.0625 (9)	
H11A	−0.2250	−0.5643	0.2315	0.075*	
H11B	−0.0586	−0.6370	0.2699	0.075*	
C12	0.0904 (4)	−0.3092 (3)	0.3061 (2)	0.0489 (7)	
H12	0.1579	−0.3061	0.2439	0.059*	
C13	−0.1055 (4)	−0.3780 (3)	0.4208 (2)	0.0474 (7)	
H13	−0.1979	−0.4325	0.4511	0.057*	
C14	−0.0133 (4)	−0.2505 (3)	0.4685 (3)	0.0513 (7)	
H14	−0.0291	−0.1992	0.5384	0.062*	
N1	0.1078 (3)	−0.2097 (3)	0.3962 (2)	0.0523 (6)	
H1A	0.175 (4)	−0.135 (4)	0.406 (3)	0.063*	
N2	−0.0387 (3)	−0.4140 (2)	0.31870 (18)	0.0419 (5)	
O1	0.5166 (2)	0.3008 (2)	−0.07003 (16)	0.0477 (5)	
H1	0.520 (4)	0.323 (4)	−0.150 (3)	0.072*	
O2	0.7193 (2)	0.1627 (2)	−0.10967 (16)	0.0504 (5)	
O3	0.8439 (3)	−0.0099 (3)	0.05360 (19)	0.0635 (6)	
H3A	0.858 (4)	0.013 (4)	−0.0196 (15)	0.095*	
O4	0.3120 (3)	0.3311 (2)	0.32481 (17)	0.0513 (5)	
O5	0.5091 (3)	0.2577 (3)	0.48529 (18)	0.0677 (6)	
O6	0.2758 (3)	0.0777 (2)	0.4006 (2)	0.0664 (6)	
O7	0.5030 (3)	0.3928 (2)	0.71130 (18)	0.0563 (6)	
H7A	0.506 (5)	0.342 (4)	0.644 (4)	0.084*	
H7B	0.565 (5)	0.471 (5)	0.700 (3)	0.084*	
S1	0.39522 (9)	0.20854 (7)	0.37902 (5)	0.0380 (2)	

### Atomic displacement parameters (Å^2^)

**Table:**

	*U*^11^	*U*^22^	*U*^33^	*U*^12^	*U*^13^	*U*^23^
C1	0.0372 (13)	0.0356 (11)	0.0304 (13)	−0.0014 (10)	0.0101 (10)	0.0005 (9)
C2	0.0420 (15)	0.0447 (13)	0.0394 (14)	0.0077 (11)	0.0145 (12)	0.0024 (11)
C3	0.0516 (17)	0.0541 (15)	0.0497 (17)	0.0238 (13)	0.0114 (13)	0.0118 (13)
C4	0.0571 (17)	0.0473 (14)	0.0346 (14)	0.0080 (12)	0.0068 (12)	0.0105 (11)
C5	0.0408 (14)	0.0367 (12)	0.0239 (11)	−0.0020 (10)	0.0084 (10)	−0.0001 (9)
C6	0.0362 (13)	0.0355 (11)	0.0282 (12)	0.0018 (10)	0.0074 (10)	0.0017 (9)
C7	0.0391 (14)	0.0405 (12)	0.0302 (12)	−0.0023 (11)	0.0115 (11)	0.0009 (10)
C8	0.0486 (16)	0.0490 (14)	0.0329 (14)	−0.0049 (12)	0.0056 (12)	−0.0065 (11)
C9	0.0567 (18)	0.0533 (16)	0.0403 (15)	0.0168 (13)	−0.0047 (13)	0.0036 (12)
C10	0.0493 (17)	0.0607 (17)	0.0476 (17)	0.0155 (14)	0.0082 (13)	−0.0092 (13)
C11	0.083 (2)	0.0612 (18)	0.0376 (16)	−0.0217 (16)	0.0181 (15)	−0.0101 (13)
C12	0.0461 (16)	0.0579 (16)	0.0411 (15)	−0.0069 (13)	0.0142 (13)	−0.0036 (12)
C13	0.0448 (15)	0.0683 (18)	0.0301 (13)	0.0035 (13)	0.0134 (12)	0.0044 (12)
C14	0.0567 (18)	0.0588 (17)	0.0397 (15)	0.0109 (14)	0.0087 (13)	−0.0068 (13)
N1	0.0543 (16)	0.0512 (14)	0.0470 (14)	−0.0095 (11)	0.0055 (12)	−0.0062 (11)
N2	0.0443 (13)	0.0500 (12)	0.0300 (11)	−0.0033 (10)	0.0100 (9)	−0.0018 (9)
O1	0.0577 (12)	0.0605 (11)	0.0293 (10)	0.0153 (9)	0.0142 (9)	0.0116 (8)
O2	0.0561 (12)	0.0623 (11)	0.0374 (10)	0.0087 (9)	0.0237 (9)	0.0049 (9)
O3	0.0681 (14)	0.0761 (14)	0.0585 (14)	0.0311 (12)	0.0333 (12)	0.0130 (11)
O4	0.0595 (12)	0.0561 (11)	0.0436 (11)	0.0171 (9)	0.0170 (9)	0.0094 (9)
O5	0.0763 (15)	0.0925 (16)	0.0349 (11)	0.0276 (13)	−0.0058 (10)	−0.0228 (11)
O6	0.0833 (15)	0.0500 (11)	0.0726 (15)	−0.0035 (10)	0.0506 (13)	0.0020 (10)
O7	0.0863 (17)	0.0511 (12)	0.0309 (10)	0.0033 (11)	0.0104 (10)	0.0057 (9)
S1	0.0512 (4)	0.0382 (3)	0.0258 (3)	0.0028 (3)	0.0128 (3)	0.0011 (2)

### Geometric parameters (Å, °)

**Table:**

C1—C2	1.399 (4)	C10—H10	0.9300
C1—C6	1.403 (3)	C11—N2	1.474 (3)
C1—C7	1.476 (3)	C11—H11A	0.9700
C2—O3	1.343 (3)	C11—H11B	0.9700
C2—C3	1.386 (4)	C12—N1	1.313 (4)
C3—C4	1.368 (4)	C12—N2	1.316 (3)
C3—H3	0.9300	C12—H12	0.9300
C4—C5	1.396 (4)	C13—C14	1.331 (4)
C4—H4	0.9300	C13—N2	1.371 (3)
C5—C6	1.374 (3)	C13—H13	0.9300
C5—S1	1.765 (2)	C14—N1	1.353 (4)
C6—H6	0.9300	C14—H14	0.9300
C7—O2	1.224 (3)	N1—H1A	0.79 (3)
C7—O1	1.313 (3)	O1—H1	0.93 (4)
C8—C9	1.376 (4)	O3—H3A	0.87 (2)
C8—C10	1.378 (4)	O4—S1	1.4444 (19)
C8—C11	1.505 (4)	O5—S1	1.441 (2)
C9—C10^i^	1.379 (4)	O6—S1	1.442 (2)
C9—H9	0.9300	O7—H7A	0.88 (4)
C10—C9^i^	1.379 (4)	O7—H7B	0.81 (4)
			
C2—C1—C6	119.0 (2)	N2—C11—C8	112.1 (2)
C2—C1—C7	119.7 (2)	N2—C11—H11A	109.2
C6—C1—C7	121.3 (2)	C8—C11—H11A	109.2
O3—C2—C3	117.2 (2)	N2—C11—H11B	109.2
O3—C2—C1	122.7 (2)	C8—C11—H11B	109.2
C3—C2—C1	120.1 (2)	H11A—C11—H11B	107.9
C4—C3—C2	120.3 (2)	N1—C12—N2	108.4 (2)
C4—C3—H3	119.9	N1—C12—H12	125.8
C2—C3—H3	119.9	N2—C12—H12	125.8
C3—C4—C5	120.5 (2)	C14—C13—N2	107.2 (2)
C3—C4—H4	119.8	C14—C13—H13	126.4
C5—C4—H4	119.8	N2—C13—H13	126.4
C6—C5—C4	119.8 (2)	C13—C14—N1	107.1 (2)
C6—C5—S1	121.96 (19)	C13—C14—H14	126.5
C4—C5—S1	118.19 (18)	N1—C14—H14	126.5
C5—C6—C1	120.3 (2)	C12—N1—C14	109.2 (2)
C5—C6—H6	119.8	C12—N1—H1A	126 (3)
C1—C6—H6	119.8	C14—N1—H1A	124 (3)
O2—C7—O1	122.8 (2)	C12—N2—C13	108.1 (2)
O2—C7—C1	122.1 (2)	C12—N2—C11	126.2 (2)
O1—C7—C1	115.1 (2)	C13—N2—C11	125.7 (2)
C9—C8—C10	118.8 (2)	C7—O1—H1	108 (2)
C9—C8—C11	120.3 (3)	C2—O3—H3A	128.1 (11)
C10—C8—C11	120.9 (3)	H7A—O7—H7B	100 (4)
C8—C9—C10^i^	120.7 (3)	O5—S1—O6	111.68 (15)
C8—C9—H9	119.6	O5—S1—O4	112.97 (13)
C10^i^—C9—H9	119.6	O6—S1—O4	112.13 (13)
C8—C10—C9^i^	120.5 (3)	O5—S1—C5	105.30 (12)
C8—C10—H10	119.8	O6—S1—C5	106.67 (11)
C9^i^—C10—H10	119.8	O4—S1—C5	107.55 (11)
			
C6—C1—C2—O3	−180.0 (2)	C9—C8—C10—C9^i^	0.2 (5)
C7—C1—C2—O3	−0.3 (4)	C11—C8—C10—C9^i^	179.6 (3)
C6—C1—C2—C3	1.0 (4)	C9—C8—C11—N2	110.3 (3)
C7—C1—C2—C3	−179.3 (2)	C10—C8—C11—N2	−69.2 (4)
O3—C2—C3—C4	−179.8 (3)	N2—C13—C14—N1	0.0 (3)
C1—C2—C3—C4	−0.8 (4)	N2—C12—N1—C14	−0.3 (4)
C2—C3—C4—C5	−0.3 (4)	C13—C14—N1—C12	0.2 (4)
C3—C4—C5—C6	1.1 (4)	N1—C12—N2—C13	0.3 (3)
C3—C4—C5—S1	−179.0 (2)	N1—C12—N2—C11	179.9 (3)
C4—C5—C6—C1	−0.8 (4)	C14—C13—N2—C12	−0.2 (3)
S1—C5—C6—C1	179.28 (17)	C14—C13—N2—C11	−179.8 (3)
C2—C1—C6—C5	−0.2 (4)	C8—C11—N2—C12	−21.6 (5)
C7—C1—C6—C5	−179.8 (2)	C8—C11—N2—C13	157.9 (3)
C2—C1—C7—O2	−0.5 (4)	C6—C5—S1—O5	−128.2 (2)
C6—C1—C7—O2	179.2 (2)	C4—C5—S1—O5	52.0 (2)
C2—C1—C7—O1	179.2 (2)	C6—C5—S1—O6	113.1 (2)
C6—C1—C7—O1	−1.2 (3)	C4—C5—S1—O6	−66.8 (2)
C10—C8—C9—C10^i^	−0.2 (5)	C6—C5—S1—O4	−7.4 (2)
C11—C8—C9—C10^i^	−179.6 (3)	C4—C5—S1—O4	172.70 (19)

Symmetry codes: (i) −*x*, −*y*−1, −*z*.

### Hydrogen-bond geometry (Å, °)

**Table:**

*D*—H···*A*	*D*—H	H···*A*	*D*···*A*	*D*—H···*A*
O1—H1···O7^ii^	0.93 (4)	1.68 (4)	2.593 (3)	168 (3)
O3—H3A···O2	0.87 (2)	2.04 (2)	2.591 (3)	121 (3)
O3—H3A···O3^iii^	0.87 (2)	2.46 (2)	2.883 (4)	111 (2)
O7—H7B···O4^iv^	0.81 (4)	1.93 (4)	2.741 (3)	173 (4)
O7—H7A···O5	0.88 (4)	1.93 (4)	2.799 (3)	172 (4)
N1—H1A···O6	0.79 (3)	1.95 (3)	2.707 (3)	160 (3)
C4—H4···O6^v^	0.93	2.51	3.411 (4)	164
C12—H12···O2^vi^	0.93	2.22	3.035 (3)	146
C14—H14···O6^vii^	0.93	2.52	3.219 (4)	132
C3—H3···Cg1^viii^	0.93	2.97 (1)	3.889 (3)	170

Symmetry codes: (ii) *x*, *y*, *z*−1; (iii) −*x*+2, −*y*, −*z*; (iv) −*x*+1, −*y*+1, −*z*+1; (v) −*x*+1, −*y*, −*z*+1; (vi) −*x*+1, −*y*, −*z*; (vii) −*x*, −*y*, −*z*+1; (viii) *x*+1, *y*, *z*.
